# Sexual health is a priority for African family medicine and primary care research

**DOI:** 10.4102/phcfm.v17i1.5302

**Published:** 2025-12-12

**Authors:** Deidre Pretorius

**Affiliations:** 1 Department of Family Medicine and Primary Care, School of Clinical Medicine, Division of Family Medicine, University of the Witwatersrand, Johannesburg, South Africa

Perhaps one day people will wonder at this. They will not be able to understand how a civilisation so intent on developing enormous instruments of production and destruction found the time and the infinite patience to inquire so anxiously concerning the actual state of sex.^1^

Why is sex then so important?

Health services deal mostly with the adverse consequences of sexual behaviour such as unintended pregnancy, sexually transmitted infections (STIs), human immunodeficiency virus (HIV) and sexual violence. Sexual problems are also a biomarker for cardiovascular and metabolic diseases and a risk factor for poor quality of life.^2,3^ We also know that chronic stress leads to maladaptive physiological sequelae that contribute to sexual dysfunction and disorders.^3^ Sexual health challenges are also associated with depression and other adverse psychosocial conditions such as gender-based violence.^2,4^ Sexual development and sexual challenges throughout life can influence the development of sexual concepts that both predict sexual behaviours and sexual health outcomes.^5,6^

On a community or societal basis, sociosexual matters often trigger stigmatisation, discrimination and prejudice towards sexual minority groups (LGBTQIA+ [lesbian, gay, bisexual, transgender, queer or questioning, intersex, and asexual, +]) – sexual orientation and identities that fall outside of heterosexual and cisgender norms.^7,8^ Research indicates that these minority groups often have poorer health outcomes.^9,10^ In sub-Saharan Africa, children and adolescents are particularly challenged by reproductive health issues such as genital mutilation, child marriages, teenage pregnancies and low contraceptive coverage.^11^ More recently, online cybersex and involuntary celibacy contribute to the sexual realities that people face daily.^12^

Researchers often look at sexuality or sexual well-being using a public health lens. The public health model has four pillars, namely, sexual health, sexual pleasure, sexual well-being and sexual justice.^13^ It seems sex, sexuality and sexual orientation are often portrayed as covert issues and not spoken of. The global notion is to counter the negative perceptions of sex and sexuality with a sex-positive approach, recognising cultural diversity in sexual practices and the various ways personal perceptions and preferences shape the meaning of sex and sexuality.^7,14^

Research exploring the same phenomenon often has inconsistent findings due to methodological differences and sociosexual restrictions.^13,15,16^ Reporting can be limited by cultural concepts or terminology aligned with Eurocentric assessment tools.^13,15,16^ Personal prejudice and legislation contribute to moral judgements, which lead to a lesser or greater flexibility regarding sex and sexuality or sexual health help-seeking in societies.^8^ All these differences make it difficult to advance the science of sexual health.

Research in scholarly publications is the window to science. An analysis of 18 696 publications on the Sustainable Development Goals (SDG) found that only 18% came from Africa.^17^ The third SDG on good health and well-being was the main focus for African researchers. Within this, the work on sexual and reproductive health services (target 7) dealt with STIs, HIV and family planning. The least researched domain was SDG 5 on gender equality.^17^ There is a need for a greater emphasis on research regarding sexual pleasure, sexual justice, sexual rights, sexual self-esteem and respect and self-determination in personal sexual well-being.^13,14^ Africa has unique cultural context-specific socio-cultural experiences and views on sexual practices, sexual health and well-being and sexual ethics and justice.

There is a new awareness about sexual health and well-being in Africa. Organisations such as the Southern African Sexual Health Association (SASHA) and the African Society for Sexual Medicine (ASSM) contribute to the training of sexual health in Africa. Furthermore, the development of the Higher Diploma in Sexual Health and HIV Medicine of the College of Family Physicians of South Africa (H Dip Sexual Health and HIV Med[SA]) presents a new qualification opportunity for interested professionals. The *African Journal of Primary Health Care and Family Medicine* has a specific section dedicated to sexual health and is interested in publishing more on this topic and related issues. If we publish what we investigate and know, we can improve sexual health outcomes in Africa by capacitating health care workers in primary care to manage the spectrum of sexual health better.

The author declares that they have no financial or personal relationships that may have inappropriately influenced them in writing this article. The author, Deidre Pretorius, serves as a section editor of this journal. The author has no other competing interests to declare.

## References

[1] Foucault M. The actual state of sex. Lapham’s Q [serial online]. 2009 [cited 2024 Nov 27];2(1). Available from: https://www.laphamsquarterly.org/eros/actual-state-sex

[2] Sadovsky R, Nusbaum M. Sexual health inquiry and support is a primary care priority. J Sex Med. 2006;3(1):3–11. 10.1111/j.1743-6109.2005.00193.x 16409213

[3] Rosati F, Williams DP, Juster RP, Thayer JF, Ottaviani C, Baiocco R. The cardiovascular conundrum in ethnic and sexual minorities: A potential biomarker of constant coping with discrimination. Front Neurosci. 2021;15:619171. 10.3389/fnins.2021.619171 34093107 PMC8170077

[4] World Health Organization. Violence against women [homepage on the Internet]. 2024 [cited 2025 Nov 07]. Available from: https://www.who.int/health-topics/violence-against-women

[5] Potki R, Ziaei T, Faramarzi M, Moosazadeh M, Shahhosseini Z. Bio-psycho-social factors affecting sexual self-concept: A systematic review. Electron Physician. 2017;9(9):5172–5178. 10.19082/5172 29038693 PMC5633209

[6] Vasilenko SA. Sexual behavior and health from adolescence to adulthood: Illustrative examples of 25 years of research from add health. J Adolesc Health Off Publ Soc Adolesc Med. 2022;71(6 Suppl):S24–S31. 10.1016/j.jadohealth.2022.08.014 PMC9890380 36404016

[7] Williams DJ, Prior E, Wegner J. Resolving social problems associated with sexuality: Can a ‘Sex-Positive’ approach help?. Soc Work. 2013;58(3):273–276. 10.1093/sw/swt024 24032308

[8] Atari M, Lai MHC, Dehghani M. Sex differences in moral judgements across 67 countries. Proc R Soc B Biol Sci. 2020;287(1937):20201201. 10.1098/rspb.2020.1201 PMC7661301 33081618

[9] Bass B, Nagy H. Cultural competence in the care of LGBTQ patients [homepage on the Internet]. Treasure Island: StatPearls Publishing; 2025. 33085323

[10] Seretlo RJ, Smuts H, Mokgatle MM. Challenges in sexual and reproductive healthcare access for queer people in Gauteng, South Africa. Afr J Prim Health Care Fam Med. 2024;16(1): 4726. 10.4102/phcfm.v16i1.4726

[11] Melesse DY, Mutua MK, Choudhury A, et al. Adolescent sexual and reproductive health in sub-Saharan Africa: Who is left behind?. BMJ Glob Health. 2020;5(1):e002231. 10.1136/bmjgh-2019-002231 PMC7042602 32133182

[12] O’Malley RL, Holt K, Holt TJ. An exploration of the involuntary celibate (Incel) subculture online. J Interpers Violence. 2022;37(7–8):NP4981–NP5008. 10.1177/0886260520959625 32969306

[13] Mitchell KR, Lewis R, O’Sullivan LF, Fortenberry JD. What is sexual wellbeing and why does it matter for public health? Lancet Public Health. 2021;6(8):e608–e613. 10.1016/S2468-2667(21)00099-2 34166629 PMC7616985

[14] Ford JV, Corona Vargas E, Finotelli Jr. I, et al. Why pleasure matters: Its global relevance for sexual health, sexual rights and wellbeing. Int J Sex Health. 2019;31(3):217–230. 10.1080/19317611.2019.1654587

[15] Morris LH. How school dictionaries treat human reproductive organs, and recommendations for South African primary school dictionaries [homepage on the Internet]. Lexikos. 2022 [cited 2025 Nov 07]. Available form: https://journals.co.za/doi/10.5788/32-3-1734

[16] Ntombana L, Humbe BP, Humbe E. Body rituals as a series of Indigenous Religious Knowledge Systems (IRKS) in advancing sexual reproductive health among Indigenous People of Zimbabwe. Afr J Relig Philos Cult. 2025;6(2):233–254. 10.31920/2634-7644/2025/v6n2a13

[17] Sweileh WM. Bibliometric analysis of scientific publications on ‘sustainable development goals’ with emphasis on ‘good health and well-being’ goal (2015–2019). Glob Health. 2020;16(1):68. 10.1186/s12992-020-00602-2 PMC7385333 32723366

